# Supplementary material for: Coexisting Nodular Sclerosis Hodgkin Lymphoma and Kimura’s Disease: A Case Report and Literature Review
Source: Int J Mol Sci. 2023 Apr 21;24(8):7666. doi: 10.3390/ijms24087666 (PMC10146151; doi:10.3390/ijms24087666)
Supplement: Supplementary file 1 [file ijms-24-07666-s001.zip › ijms-2288632-supplementary.pdf]

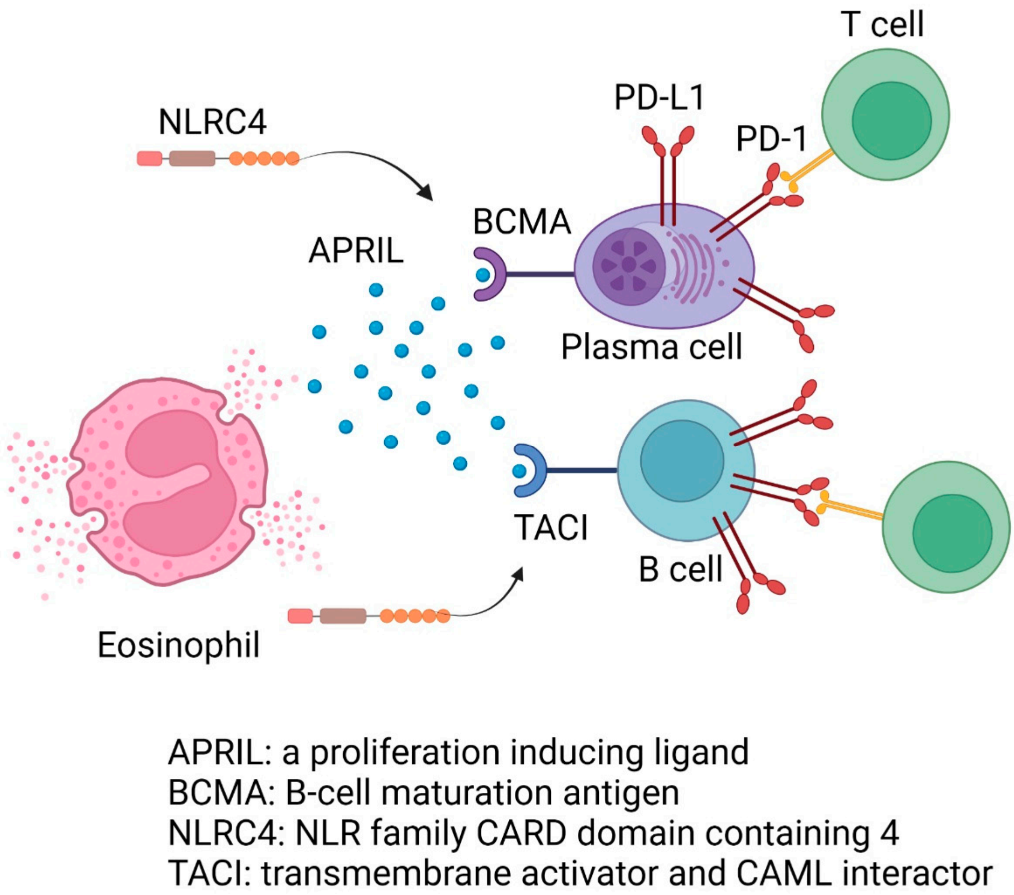

Supplemental Figure S1. The biological role of eosinophils in upregulating the expression of programmed death-ligand 1 (PD-L1).
